# On the onset of surface condensation: formation and transition mechanisms of condensation mode

**DOI:** 10.1038/srep30764

**Published:** 2016-08-02

**Authors:** Qiang Sheng, Jie Sun, Qian Wang, Wen Wang, Hua Sheng Wang

**Affiliations:** 1 School of Engineering and Materials Science, Queen Mary University of London, London E1 4NS, UK; 2 Institute of Engineering Thermophysics, Chinese Academy of Sciences, Beijing 100190, China; 3 Department of Astronomy, University of Maryland, College Park, MD 20742, USA

## Abstract

Molecular dynamics simulations have been carried out to investigate the onset of surface condensation. On surfaces with different wettability, we snapshot different condensation modes (no-condensation, dropwise condensation and filmwise condensation) and quantitatively analyze their characteristics by temporal profiles of surface clusters. Two different types of formation of nanoscale droplets are identified, i.e. the formations with and without film-like condensate. We exhibit the effect of surface tensions on the formations of nanoscale droplets and film. We reveal the formation mechanisms of different condensation modes at nanoscale based on our simulation results and classical nucleation theory, which supplements the ‘classical hypotheses’ of the onset of dropwise condensation. We also reveal the transition mechanism between different condensation modes based on the competition between surface tensions and reveal that dropwise condensation represents the transition states from no-condensation to filmwise condensation.

Vapor condensation on a cooled surface is conventionally categorized as either dropwise condensation (DWC) or filmwise condensation (FWC). In dropwise condensation, the surface covered by droplets of different sizes is not completely wetted by condensate. Many natural phenomena, for example, condensation on lotus leaves^1^ and butterfly wings^2^ show dropwise condensation mode while condensation on metal surfaces often shows filmwise condensation mode^3^. Dropwise condensation can be obtained on micro/nanostructured surfaces or chemically modified surfaces with lowered surface free energy and desired wettability^4^. Wettability, typically characterized by the contact angle (*θ*), is generally described as either hydrophilic (*θ* < 90°) or hydrophobic (*θ* > 90°)^5^. Particularly, with even larger *θ* than the naturally achievable maximum (*θ* ≈ 120°), the wettability is further termed as ultra-hydrophobic (*θ* > 120°) and super-hydrophobic (*θ* > 150°)^5^,^6^.

In dropwise condensation, a typical cycle of the evolution of droplets usually consists of formation, growth, coalescence and departure. The sizes of these droplets are observed at the scales from micrometer, millimeter to centimeter. Their size distribution and development have also been widely investigated^7^,^8^,^9^,^10^. However, the formation of initial nanoscale droplets, on which this report is focused, has not yet been fully understood. Since dropwise condensation was recognized in 1930^11^, the formation mechanism of the initial droplets has been explained from different angles^12^,^13^,^14^,^15^,^16^,^17^,^18^,^19^,^20^,^21^,^22^ and two ‘hypotheses’ were proposed, namely ‘the hypothesis of film-rupture’^18^ and ‘the hypothesis of specific nucleation sites’^21^. The former suggests that a thin condensate film forms on the surface and then the film ruptures into droplets due to the effect of surface tensions when the film reaches a critical thickness^18^,^19^. The latter suggests that nuclei directly initiate at specific nucleation sites, such as pits, caves or grooves^20^,^21^,^22^. The majority studies seem to support ‘the hypothesis of specific nucleation sites’^15^, however, the understanding of fundamental physics of the formation of initial droplets is still incomplete.

Dropwise condensation fundamentally originates from nucleation process. The concept of molecular clustering has been introduced to explain the formation mechanism of initial droplets^23^. The nucleation process has been investigated using the Monte Carlo (MC) simulation^24^,^25^ and the molecular dynamics (MD) simulation^26^,^27^,^27^,^28^,^29^,^30^,^31^,^32^,^33^. This includes the distribution of critical cluster size^27^, free energy barrier of cluster formation^28^ and effect of surface free energy^32^,^33^. Although these investigations have shed some light on the formation mechanism of clusters, few studies concern how the initial droplets appear after the formation of clusters. In this report, nucleation processes are investigated using MD simulation in a relatively large timescale to exhibit how the initial droplets develop. We further reveal the formation mechanisms of different condensation modes and the transition mechanism between them.

## Results

**Figure 1:**
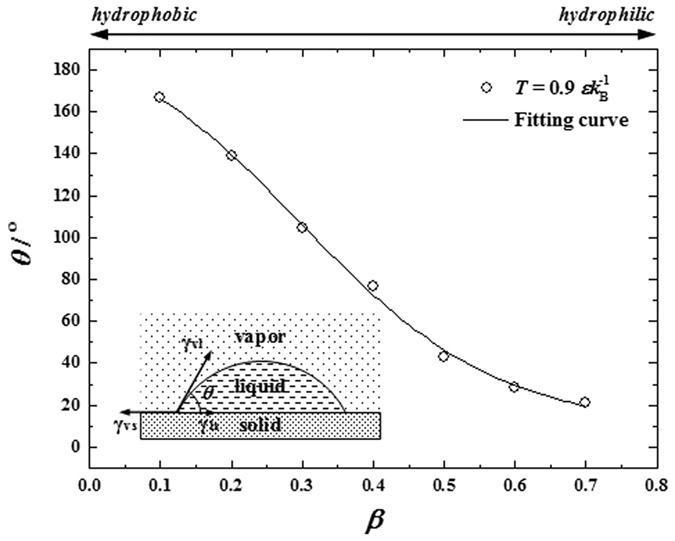
Relation between contact angle (*θ*) and fluid-solid bonding strength parameter (*β*) at 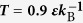.

We apply MD simulation to condensation of Lennard-Jones (L-J) vapor on cooled solid surfaces. The saturated vapor and solid surface are in thermodynamic equilibrium at 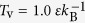 before the solid surface is suddenly cooled to 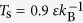 (Δ*T* = *T*_v_ − *T*_s_) at *t* = 0 *τ, τ* being the time scale. The fluid-solid interaction is also described by the L-J potential function with a fluid-solid bonding strength parameter *β*, representing the relative strength of fluid-solid interaction compared to the fluid-fluid interaction. The relative surface free energy is set at different levels by adjusting the value of *β*. On increasing *β*, the relative surface free energy increases. The values of *θ* on surfaces with different *β* are obtained using the method of density contour of droplets^34^. As shown in Fig. 1, with relative surface free energy increasing, wettability is promoted and *θ* decreases. By data-fitting, we acquired the correlation between *θ* and *β* as *θ* = *g*(*β*) = 9.22 + 195.50/(1 + exp((*β−*0.30)/0.14)). We performed MD simulations for various values of fluid-solid bonding strength.

**Figure 2:**
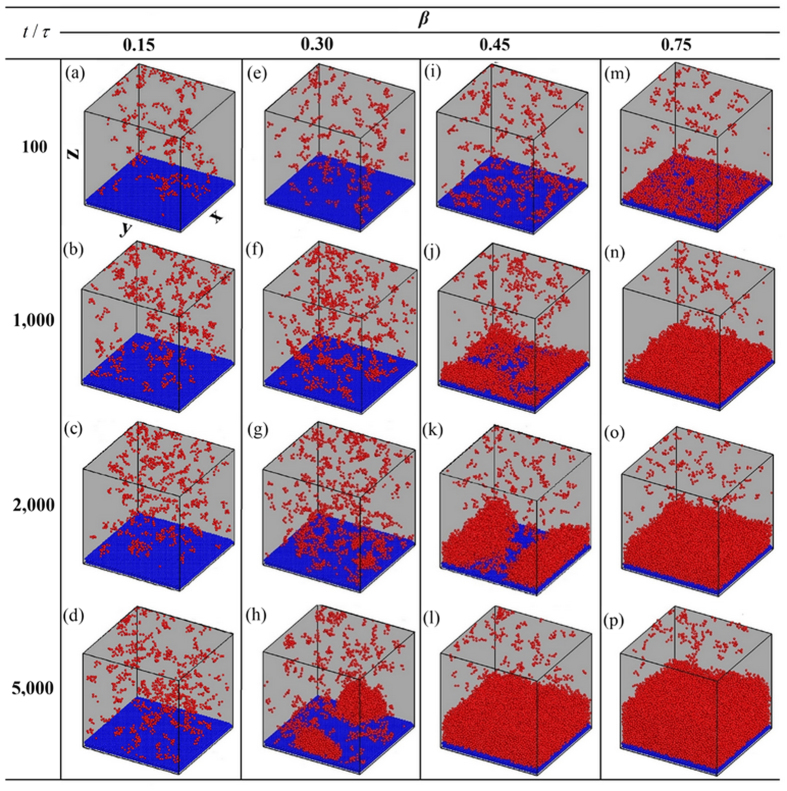
Transient snapshots of all the clusters. The surfaces are at the same temperature 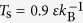 but with different fluid-solid bonding parameters: *β* = 0.15, 0.30, 0.45 and 0.75 (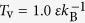, 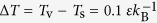). The system size is *l*_*x*_ × *l*_*y*_ × *l*_*z*_ = 47.2 *σ* × 48.0 *σ* × 92.0 *σ*. The fluid molecules not in any cluster are not shown while those in the clusters are shown in red. The solid molecules are shown in blue. The snapshots are taken at different times: 100 *τ*, 1000 *τ*, 2000 *τ* and 5000 *τ*. Only the lower half of simulation system is shown.

**Figure 3:**
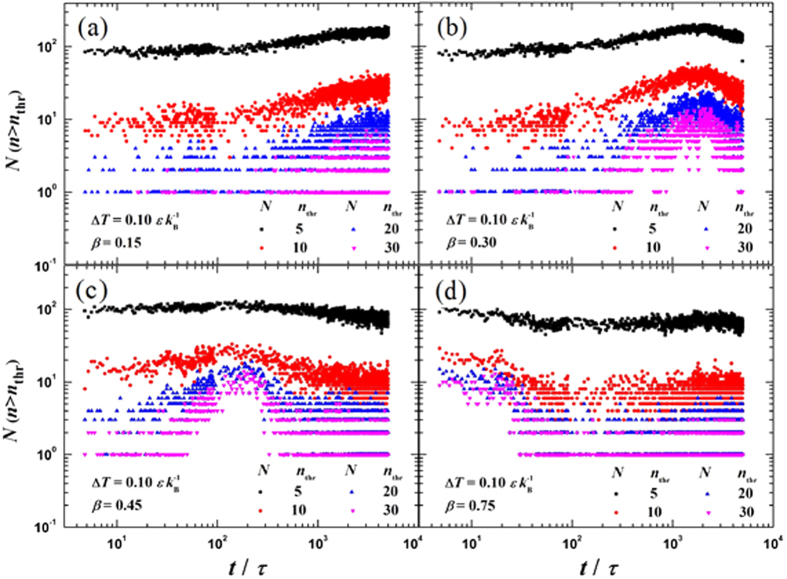
Evolution of the number (*N*) of clusters with the size larger than the threshold number of molecules (*n*_thr_). The surfaces are at the same temperature 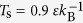 but with different fluid-solid bonding parameters: (**a**) *β* = 0.15; (**b**) *β* = 0.30; (**c**) *β* = 0.45 and (**d**) *β* = 0.75 (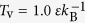, 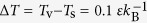).

**Figure 4:**
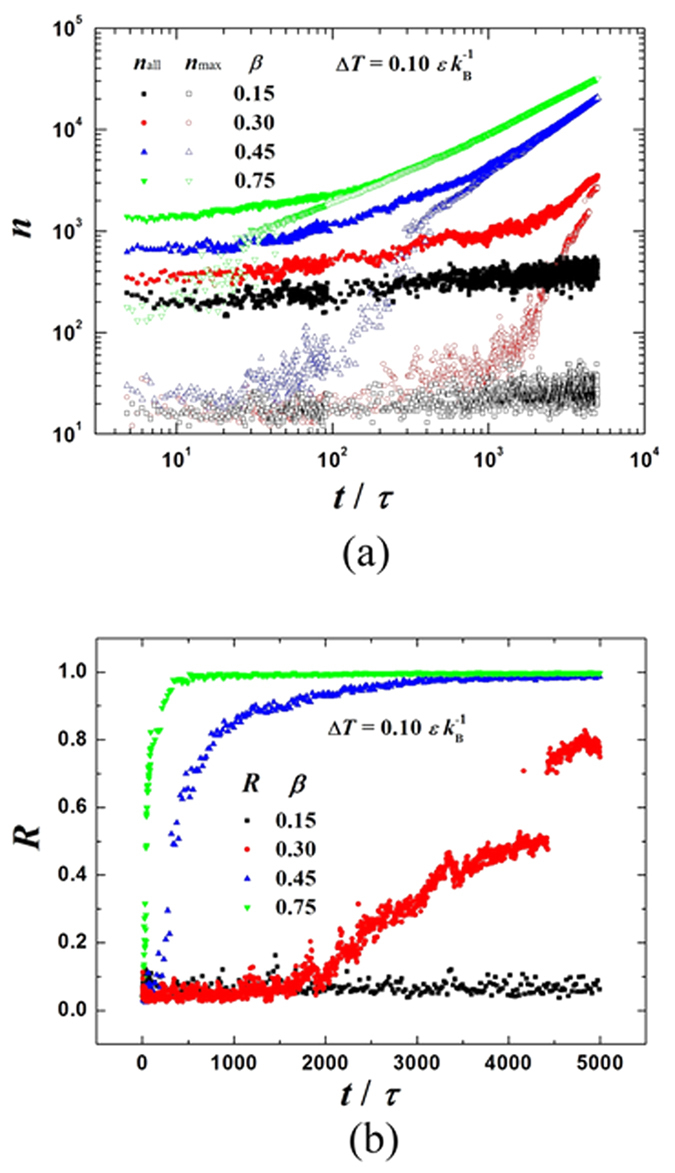
Time evolutions. (**a**) Evolutions of the number of molecules in the maximum size surface cluster (*n*_max_) and the number of molecules in all surface clusters (*n*_all_); (**b**) The ratio (*R*) of *n*_max_ over *n*_all_. The surfaces are at the same temperature 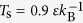 but with different fluid-solid bonding parameters: *β* = 0.15, 0.30, 0.45 and 0.75 (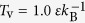, 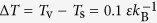).

Figure 2 shows the transient snapshots (*t* = 100 *τ*, 1000 *τ*, 2000 *τ* and 5000 *τ*) for the cases with four different bonding strength parameters: *β* = 0.15, 0.30, 0.45 and 0.75. To quantitatively analyze the nucleation process, we monitor the profile and behavior of clusters. Based on Stillinger’s definition^35^, any two molecules separated by less than a certain bonding distance *r*_b_ (*r*_b_ = 1.5 *σ*) are regarded to belong to a cluster. We measure the size of a cluster in terms of its number of molecules *n* and define the cluster as an *n*-cluster. As shown in Fig. 3, to obtain the distribution of cluster size we plot the evolution of the number (*N*) of clusters having molecules more than a certain threshold *n*_thr_, i.e. *n* > *n*_thr_. In the present work, we take the value of *n*_thr_ to be 5, 10, 20 and 30, respectively. Moreover, if the smallest distance between a cluster and the surface is less than 3.0 *σ*, the cluster is defined as a surface cluster. The evolutions of the number of molecules in all surface clusters *n*_all_ and the number of molecules in the maximum size surface cluster *n*_max_ are shown in Fig. 4a. The ratio *R* (*R* = *n*_max_/*n*_all_) is illustrated in Fig. 4b. When *R* approaches unity, it indicates that almost all surface clusters are connected as a droplet or a film.

On the super-hydrophobic surface with *β* = 0.15 (*θ* ≈ 153°), as shown in Fig. 2a–d, no condensation occurs. Although the number of clusters increases after the surface is cooled (Fig. 3a), *n*_max_ remains less than 50 (Fig. 4a), indicating that almost no cluster ever survives and evolves into a droplet (see Supplementary Video S1).

On the surface with *β* = 0.30 (*θ* ≈ 105°), as shown in Fig. 2e–h, the clusters form discretely (Fig. 2e) and randomly deposit on the surface (Fig. 2f). Both the size and number of clusters increase with time (see Fig. 3b), but *n*_max_ increases gradually and its value is less than 100 until *t* = 1000 *τ*, see Fig. 4a. Some clusters are able to migrate on the surface and coalesce with other clusters. Note that the coalescence of large clusters leads to a sudden increase in the value of *n*_max_, as shown by the stepwise evolution of *R* in Fig. 4b. If a cluster becomes large enough to possibly overcome the free energy barrier of nucleation (see below), it forms a nucleus. Some nuclei continue growing up towards nanoscale droplets while others downsize and fail. This diverse evolution is evidenced by the fluctuation in *n*_max_, as shown in Fig. 4a. Nevertheless, *n*_max_ keeps increasing while the surviving nuclei continue evolving towards nanoscale droplets (Fig. 2g). After numerous coalescences, only one primary droplet is observable (see Fig. 2h). *θ* of this primary droplet is ca. 100° (ca. 105° in Fig. 1). This primary droplet continues growing up by absorbing the clusters and molecules nearby. It is found that an initial droplet develops through three overlapping stages, namely the formation of clusters, generation of nuclei and emergence of nanoscale droplets (see Supplementary Video S2).

**Figure 5:**
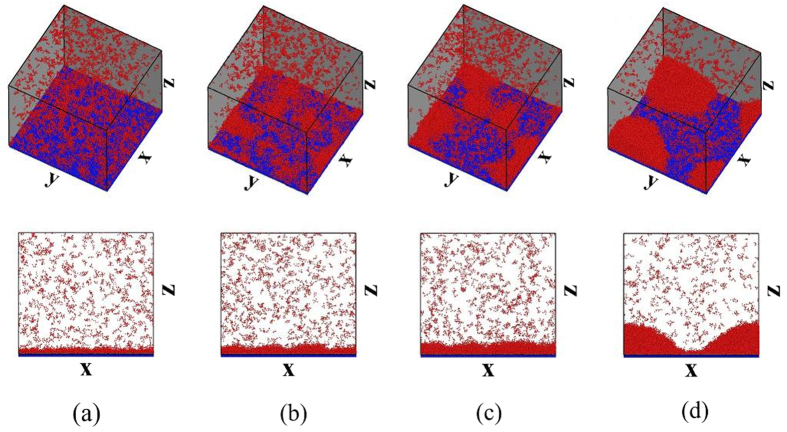
Transient snapshots of all the clusters. The surfaces is at the temperature 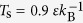 and with the wettability *β* =  0.45 (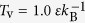, 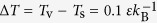). The simulation system size is *l*_*x*_ × *l*_*y*_ × *l*_*z*_ = 92.7 *σ* × 93.0 *σ* × 183.0 *σ*. The fluid molecules not in any cluster are not shown while those in the clusters are shown in red. The solid molecules are shown in blue. The snapshots are taken at different times: (**a**) 400 *τ*; (**b**) 800 *τ*; (**c**) 1000 *τ* and (**d**) 3000 *τ*. Only the lower half of simulation system is shown.

On the surface with *β* = 0.45 (*θ* ≈ 60°), as shown in Fig. 2i–l, the thermal resistance of the liquid-solid interface decreases due to stronger fluid-solid interaction^36^,^37^. Consequently, more clusters are seen to quickly and discretely deposit on the surface (see Fig. 2i) and both the number and size of clusters increase rapidly (see Figs 3c and 4a). Most of the surface clusters are connected at *t* = 1000 *τ* (*R* ≈ 0.85 in Fig. 4b), indicating a film-like condensate, despite that part of the surface area is not covered by the condensate (see Fig. 2j). Then, vapor molecules keep condensing continuously and directly into the existing film-like condensate. No appreciable stepwise evolution of *R* is seen in Fig. 4b. Afterwards, the film-like condensate contracts and ruptures into nuclei and then forms a cap-shaped droplet (see Fig. 2k). *θ* of the cap-shaped droplet is ca. 60° (ca. 60° in Fig. 1). Finally, the droplet is pulled into a film due to the finite system size under a periodic boundary condition (see Supplementary Video S3). To check that the evolution is not affected by the system size, we repeated the simulation on this surface (*β* = 0.45) but the surface area is three times larger. The results are shown in Fig. 5. A similar film-like condensate firstly emerges and then contracts and ruptures locally into several, not completely separated nuclei. These nuclei grow up with continuous supplement of vapor molecules, then merge with other nuclei in the vicinity, and eventually develop into one large droplet with *θ* ≈ 60° (see Supplementary Video S4).

On the surface with *β* = 0.75 (*θ* ≈ 16°), as shown in Fig. 2m–p, filmwise condensation is observed. The fluid-solid interaction is sufficiently strong so that numerous clusters form randomly on the surface immediately when the surface is cooled. The number of clusters is large enough so that the condensate quickly covers the whole surface (see Fig. 2m) and develops into a film (*R* ≈ 1.0 since about 500 *τ* in Fig. 4b). The film continues to grow thicker (see Fig. 2n–p) and no condensate film rupturing is seen. Therefore, a filmwise condensation is identified. Note that the initial clusters coalesce into a film-like condensate in both the cases for *β* = 0.45 and 0.75, however, the difference is that the film-like condensate in the former case contracts and ruptures into several nuclei and evolves into a large droplet while the film-like condensate in the latter case develops into a complete condensate film eventually (see Supplementary Video S5).

## Discussion

Different condensation modes have been exhibited above and the mechanisms will be explained below based on the classical nucleation theory (CNT)^38^ and our simulation results.

According to CNT, the minimal work required for an *n*-cluster to form is equal to the change of the Gibbs free energy Δ*G*(*n*), which is called the Gibbs free energy of cluster formation, as^39^


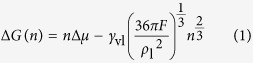


where Δ*μ* is the difference between the chemical potentials of vapor bulk *μ*_v_ and liquid bulk *μ*_l_ (Δ*μ* = *μ*_v_ − *μ*_l_), *γ*_vl_ is the vapor-liquid surface tension, *ρ*_l_ is the density of droplet and *F* = *f*(*θ*) is the Fletcher factor with the value between 0 and 1 as *θ* varies from 0° to 180°. The Fletcher factor generally accounts for the geometric effect due to different wetting status. For a droplet on a flat surface, *F* = *f*(*θ*) = (2−3cos *θ* + cos^3^ *θ*)/4^38^. On increasing *n*, Δ*G*(*n*) first increases and then decreases after a maximum ΔG^*^ is reached. ΔG^*^ is called the Gibbs free energy barrier of cluster formation, as^39^


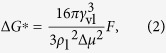


and the corresponding *n* is called the critical number of molecules of cluster formation (*n*_c_), as^39^


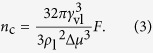


Theoretically, only when the Gibbs free energy barrier of cluster formation is overcome and the critical number of molecules of cluster formation is exceeded could a newly-formed cluster survive. In other words, ΔG^*^ and *n*_c_ quantify the difficulty of cluster formation and thus the tendency of condensation to occur. For simplification, Eqs (2) and (3) could be reduced as


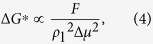



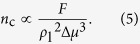


**Figure 6:**
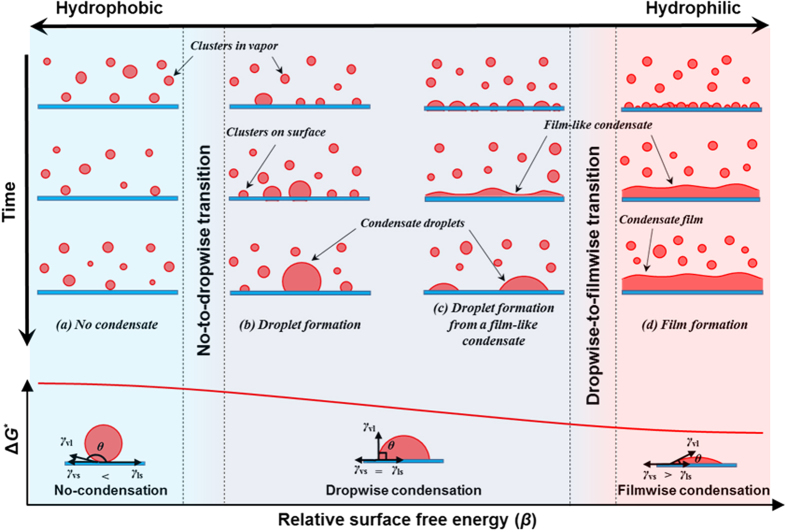
Schematic presentation of the formation and transition mechanisms of surface condensation.

Basically, *γ*_vl_ is upon the nature of the fluids and primarily determined by the thermodynamic state at vapor-liquid interface. Since throughout all the present simulations the vapor bulk is well confined at the saturation state of 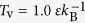 and solid temperature is constant at 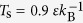, the thermodynamic state at the vapor-liquid interface could be regarded as constant and so does *γ*_vl_. Previous MD studies have clearly revealed that increasing *β* significantly decreases the liquid-solid interfacial thermal resistance *R*_t_^36^,^37^, which reduces the temperature jump *T*_j_ between solid and liquid and thus lowers the liquid bulk temperature *T*_l_ in the simulations. It is known that *ρ*_l_ increases with decreasing *T*_l_. Meanwhile, decreasing *T*_l_ enlarges Δ*μ* because *μ*_v_ is fixed due to constant thermodynamic state of vapor bulk but *μ*_l_ decreases due to higher stability at lower temperature for liquid bulk. Considering the relations, we find that increasing *β* tends to reduce ΔG^*^ and *n*_c_ according to the correlations as *β* ↑ → *R*_t_ ↓ → *T*_j_ ↓ → *T*_l_ ↓ → *ρ*_l_ ↑ & Δ*μ* ↑ → ΔG^*^ ↓ & *n*_c_ ↓. On the other hand, the simulation results clearly illustrate that *θ* decreases with increasing *β* (see Fig. 1) and we know that *F* = *f*(*θ*) is an increasing function of *θ* for the case of a droplet on a flat surface. Considering the relations, we readily obtain the correlations as *β* ↑ → *θ* ↓ → *F* ↓ → ΔG^*^ ↓ & *n*_c_ ↓. Therefore, we can conclude that increasing *β* leads to decreasing ΔG^*^ and *n*_c_. According to CNT, this conclusion suggests that as the relative surface free energy increases, both the Gibbs free energy barrier and the critical number of molecules of cluster formation decrease, which eventually drives the surface condensation to occur more easily (see curve in lower panel of Fig. 6).

Figure 6 is a schematic presentation showing the formation mechanisms of no-condensation, dropwise condensation and filmwise condensation. On the surface with *β* = 0.15 (Fig. 6a), ΔG^*^ and *n*_c_ are so large that no clusters can survive and evolve into droplet or film. Therefore, no condensation is observable.

On the surface with *β* = 0.30 (Fig. 6b), ΔG^*^ and *n*_c_ decrease, which raises the probability for newly-formed surface clusters to survive. The survived surface clusters continue to grow and coalesce, which leads to the formation of nuclei and then droplets. The limited number of surface clusters can hardly form any condensate film. This droplet formation mechanism is in line with ‘the hypothesis of specific nucleation sites’^21^, except that the nuclei occurring here are triggered and located randomly on the perfectly smooth surface rather than specific nucleation sites, e.g. pits, caves or grooves.

However, on the surface with *β* = 0.45 (Fig. 6c), the formation mechanism of nanoscale droplets is significantly different. With ΔG^*^ and *n*_c_ further decreasing, the number of clusters forming and depositing on the surface is large enough to quickly generate a film-like condensate (thickness ca. several nanometers). It then contracts and ruptures into nuclei and droplet. This droplet formation mechanism is in line with ‘the hypothesis of film-rupture’^18^.

On the surface with *β* = 0.75 (Fig. 6d), much larger *β* further reduces ΔG^*^ and *n*_c_, and drives numerous clusters to immediately generate on the surface. The enhanced liquid-solid interaction makes them rest on the surface rather than condense onto a droplet. The rested clusters cover most of the surface area and readily connect to form a film-like condensate. The film-like condensate continues growing up into a complete film, identifying the condensation mode to be filmwise.

As shown in the two cases of *β* = 0.35 and 0.45, neither of the ‘classical hypotheses’ can independently describe the formation mechanisms of nanoscale droplets. In fact, either describes the specific scenario at a certain wettability. They may be complementary processes at nanoscale rather than independent as reported before^15^.

As discussed above, we find that *β* determines ΔG^*^ and *n*_c_, which quantifies the difficulty of cluster formation based on CNT (see inserts in lower panel of Fig. 6). We also find that the dynamics of surface clusters significantly affects the transition of condensation mode according to our simulation results. Essentially, the dynamics of surface clusters is closely related to the balance of surface tensions at the vapor-liquid-solid triple-phase contact-line, which is generally described by the Young’s equation (see insert in Fig. 1):


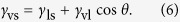


Then we have


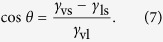


where *γ*_vs_ and *γ*_ls_ are the vapor-solid and liquid-solid surface tensions. As is stated above that *γ*_vl_ basically keeps constant throughout the present simulations. With increasing *β*, the fluid-solid interaction becomes stronger, therefore the adhesive forces between the fluids and solid transcend the cohesive forces within the fluid bulks, which leads to decreases in *γ*_vs_ and *γ*_ls_. On the other hand, the *β*-induced variation in *γ*_ls_ is always greater than that in *γ*_vs_ due to much stronger liquid-solid interaction than the vapor-solid one. i.e. *γ*_ls_ is more sensitive to *β*. Therefore, we readily have *β* ↑ → *γ*_vs_ ↓ & *γ*_ls_ ↓↓ → (*γ*_vs_−*γ*_ls_) ↑ → cos *θ*↑ → *θ* ↓. Note that there exists a critical value of *β* ensuring *θ* = 90° (e.g. *β* ≈ 3.5 in Fig. 1), indicating *γ*_vs_ = *γ*_ls_. The eventual condensation mode is in fact decided by the competition between the surface tensions (see inserts in lower panel of Fig. 6). Take the diverse evolutions of the film-like condensate in the cases of *β* = 0.45 and 0.75 for an example. The film-like condensate contracts and ruptures and dropwise condensation arises if the cohesive forces overcome the adhesive forces. Otherwise, the film-like condensate grows thicker and filmwise condensation develops. Specifically, when abundant clusters form on the surface, the surface tensions determine the eventual condensation mode through affecting the dynamics of surface clusters. If the fluid-solid interaction keeps decreasing until it is too weak to possibly generate nuclei on the surface, eventually dropwise condensation disappears and no condensation could ever occur on the surface. It is predictable that there could exist two critical values of *β*. The first critical value lies in the no-to-dropwise transition and separates no-condensation mode and dropwise condensation mode. This is essentially a threshold of surface clusters between ‘zero’ and ‘few’. The second critical value lies in the dropwise-to-filmwise transition and separates dropwise and filmwise condensation modes. This is essentially a threshold of surface clusters between ‘few’ and ‘many’. From the view of surface tension competition, dropwise condensation represents the transition states between no-condensation mode and filmwise condensation mode. The transition mechanism between different condensation modes is explicitly illustrated in lower panel of Fig. 6 in terms of relative surface free energy (*β*). Other experimental^17^,^40^,^41^,^42^ and numerical^31^,^33^ investigations also support the transition mechanism that by physically or chemically lowering the relative surface free energy, e.g. micro/nanomachining^17^,^40^,^41^, chemical coating^17^,^42^ and fluid with higher surface tension^41^, the condensation mode changes from filmwise to dropwise with visible decreasing *θ*. It is noteworthy that micro/nanomachining and chemical coating are direct resorts of lowering the absolute surface free energy while fluid with higher surface tension is the resort of increasing the cohesive fluid-fluid interaction. Equivalently, there are all resorts of lowering the relative surface free energy (*β*).

**Figure 7:**
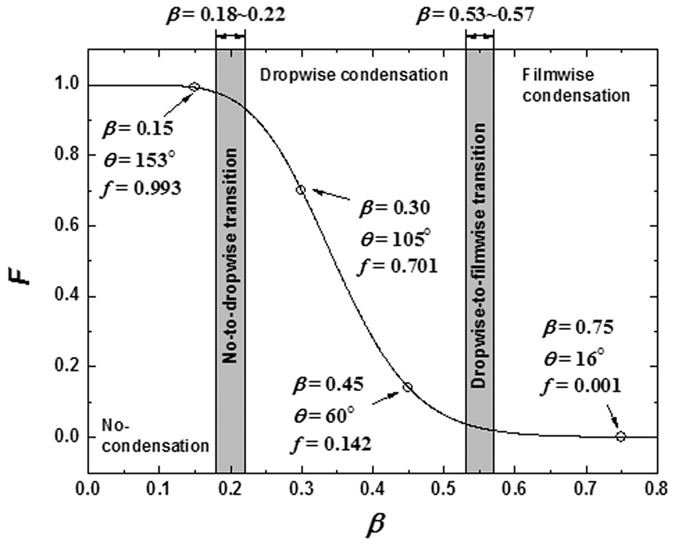
Correlation between the Flecher factor (*F*) and the fluid-solid bonding parameter (*β*).

To determine the critical values of *β* for defining different condensation modes, we borrow the Fletcher factor as the criterion. Since we already acquired the correlations of *F* = *f*(*θ*) and *θ* = *g*(*β*), we readily have the correlation between *F* and *β* (see Fig. 7). As is expected, there exist two apparent turning points (*β* ≈ 0.20 and *β* ≈ 0.55), which could be generally regarded as the critical values, dividing the range of *β* into three regions, corresponding to non-condensation, dropwise condensation and filmwise condensation. The present cases shown in Fig. 2 lie in the corresponding regions. By analyzing more simulations with a serial values of *β* in details, the critical values are further determined within *β* = 0.18~0.22 and *β* = 0.53~0.57. In summary, we present the evolutions from clusters to nucleus and nanoscale droplets or to liquid film, undergoing different condensation modes. We qualitatively examine the characteristics of different condensation modes by transient snapshots and quantitatively analyze the evolutions of the number and size of clusters. We find that the initial droplets in dropwise condensation could form in two significantly different ways depending on the relative surface free energy. We reveal the formation mechanisms of different condensation modes at nanoscale based on our simulation results and classical nucleation theory, which supplements the ‘classical hypotheses’ of the onset of dropwise condensation. We also reveal the transition mechanism between different condensation modes based on the competition between surface tensions and reveal that dropwise condensation represents the transition state from no-condensation to filmwise condensation.

## Methods

The basic simulation system size measures *l*_*x*_ × *l*_*y*_ × *l*_*z*_ = 47.2 *σ* × 48.0 *σ* × 92.0 *σ*. A larger system of 92.7 *σ* × 93.0 *σ* × 183.0 *σ* is used to examine the effect of finite system size. The fluid-fluid interaction is governed by the Lennard-Jones (L-J) potential function *φ*(*r*) = 4*ε*[(*σ*/*r*)^12^−(*σ*/*r*)^6^], where *r* is the intermolecular separation, *σ* is the length scale and *ε* is the energy scale. The function is truncated at the cut-off radius *r*_c_ = 4.0 *σ*, beyond which molecular interactions are neglected. The semi-infinite solid wall at the bottom end is represented by three layers of solid molecules forming a (111) plane of a face-centered cubic lattice with the lattice constant *σ*_s_ = 0.814 *σ*. Neighboring solid molecules are connected by Hookean springs with the constant *k* = 3249.1 *εσ*^−2 ^43. For temperature control, two extra layers of solid molecules are set below the three layers. The lower layer is stationary as a frame while the upper is governed by the Langevin thermostat 
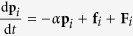
, where *α* = 168.3 *τ*^−1^ is the damping constant^44^, **p**_*i*_ is the momentum of the *i*th solid molecule; **f**_*i*_ is the sum of the forces acting on the *i*th solid molecule, **F**_*i*_ is a random force, of which each component is sampled from the Gaussian distribution with zero mean value and variance 2*αk*_B_*T*_s_/*δt* (*k*_B_ is the Boltzmann constant and *δt* = 0.002 *τ* is the time step, where 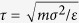 is the time scale, *m* being the mass of a fluid molecule). This technique of constant temperature control is feasible for both fluid^45^,^46^ and solid^43^,^44^. The fluid-solid interaction is also described by the L-J potential function but with a different length scale *σ*_fs_ = 0.91 *σ* and energy scale *ε*_fs_ = *βε*, where the fluid-solid bonding strength parameter *β* measures the wettability. In each simulation, the vapor molecules are uniformly arranged with the saturation density corresponding to 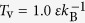. A period of 200 *τ* is allowed for the system to reach thermal equilibrium state at *T*_v_ before the surface temperature is reduced to 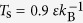 (Δ*T* = *T*_v_ − *T*_s_) at *t* = 0. Afterwards, the condensation process evolves for a period of time 5000 *τ*. Extra vapor molecules are supplied through the supply region at the top end (thickness *l*_*z*_/10) during condensation process. The molecular insertion is immediately carried out by the USHER algorithm^47^ when the average density within the supply region is lower than its initial saturation value. The temperature in the supply region is controlled at 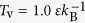 by the Langevin thermostat^45^. Therefore, the vapor bulk is maintained at the saturation state of 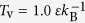 all through the simulations. Periodic boundary condition and diffuse reflection boundary condition are employed at the sides and top end, respectively. The leapfrog scheme is used for integrating the equations of motion and the cell subdivision technique is used to improve the computational efficiency^48^,^49^.

## Additional Information

**How to cite this article**: Sheng, Q. *et al*. On the onset of surface condensation: formation and transition mechanisms of condensation mode. *Sci. Rep.* **6**, 30764; doi: 10.1038/srep30764 (2016).

## Supplementary Material

Supplementary Information

Supplementary Information

Supplementary Information

Supplementary Information

Supplementary Information

Supplementary Information

## References

[b1] Gao L. C. & McCarthy T. J. The “lotus effect” explained: two reasons why two length scales of topography are important. Langmuir 22, 2966–2967 (2006). 16548542 10.1021/la0532149

[b2] Zheng Y., Gao X. F. & Jiang L. Directional adhesion of superhydrophobic butterfly wings. Soft Matter 3, 178–182 (2007). 10.1039/b612667g 32680261

[b3] Wang H. S. & Rose J. W. Film condensation in horizontal microchannels: effect of channel shape. Int. J. Therm. Sci. 45, 1205–1212 (2006).

[b4] Feng L. . Super-hydrophobic surfaces: from natural to artificial. Adv. Mater. 14, 1857–1860 (2002).

[b5] Quéré D. Wetting and roughness. Annu. Rev. Mater. Res. 38, 71–99 (2008).

[b6] Roach P., Shirtcliffe N. J. & Newton M. I. Progess in superhydrophobic surface development. Soft Matter 4, 224–240 (2008). 10.1039/b712575p 32907233

[b7] Sikarwar B. S., Khandekar S., Agrawal S., Kumar S. & Muralidhar K. Dropwise condensation studies on multiple scales. Heat Transfer Eng. 33, 301–341 (2012).

[b8] Hu H. W. & Tang G. H. Theoretical investigation of stable dropwise condensation heat transfer on a horizontal tube. Appl. Therm. Eng. 62, 671–679 (2014).

[b9] Rykaczewski K. Microdroplet growth mechanism during water condensation on superhydrophobic surfaces. Langmuir 28, 7720–7729 (2012). 22548441 10.1021/la301618h

[b10] Wier K. A. & McCarthy T. J. Condensation on ultrahydrophobic surfaces and its effect on droplet mobility: ultrahydrophobic surfaces are not always water repellant. Langmuir 22, 2433–2436 (2006). 16519435 10.1021/la0525877

[b11] Schmidt E., Schurig W. & Sellschopp W. Versuche uber die kondensation von: wasserdampf in film-und tropfenform. Tech. Mech. Thermodyn. 1, 53–63 (1930).

[b12] Mikic B. B. On mechanism of dropwise condensation. Int. J. Heat Mass Transfer 12, 1311–1323 (1969).

[b13] Umur A. & Griffith P. Mechanism of dropwise condensation. J. Heat Transfer-T ASME 87, 275–282 (1965).

[b14] Rose J. W. On the mechanism of dropwise condensation. Int. J. Heat Mass Transfer 10, 755–762 (1967).

[b15] Khandekar S. & Muralidhar K. Dropwise condensation on inclined textured surfaces (Springer, 2014).

[b16] Rose J. W. Dropwise condensation theory and experiment: a review. P. I. Mech. Eng. A-J. Pow. 216, 115–128 (2002).

[b17] Enright R., Miljkovic N., Alvarado J. L., Kim K. & Rose J. W. Dropwise condensation on micro-and nanostructured surfaces. Nanosc. Microsc. Therm. 18, 223–250 (2014).

[b18] Jacob M. Heat transfer in evaporation and condensation II. Mech. Eng. 58, 729–740 (1936).

[b19] Utaka Y. & Terachi N. Measurement of condensation characteristic curves for binary mixture of steam and ethanol vapor. Heat Transfer Jpn. Res. 24, 57–67 (1995).

[b20] Tammann G. & Boehme W. Die zahl der wassertrcpfchen bei der condensation aufverschiedenen fasten stiffen. Arm Physik 5, 77–80 (1935).

[b21] Eucken A. Energie-und stoffaustausch an grenzflächen. Naturwissenschaften 25, 209–218 (1937).

[b22] Liu T. Q., Mu C. F., Sun X. Y. & Xia S. B. Mechanism study on formation of initial condensate droplets. AIChE J. 53, 1050–1055 (2007).

[b23] Song T. Y., Lan Z., Ma X. H. & Bai T. Molecular clustering physical model of steam condensation and the experimental study on the initial droplet size distribution. Int. J. Therm. Sci. 48, 2228–2236 (2009).

[b24] Merikanto J., Vehkamäki H. & Zapadinsky E. Monte Carlo simulations of critical cluster sizes and nucleation rates of water. J. Chem. Phys. 121, 914–924 (2004). 15260623 10.1063/1.1740754

[b25] Oh K. J. & Zeng X. C. Formation free energy of clusters in vapor-liquid nucleation: a Monte Carlo simulation study. J. Chem. Phys. 110, 4471–4476 (1999).

[b26] Wedekind J., Reguera D. & Strey R. Finite-size effects in simulations of nucleation. J. Chem. Phys. 125, 214505 (2006). 17166031 10.1063/1.2402167

[b27] Yasuoka K. & Matsumoto M. Molecular dynamics of homogeneous nucleation in the vapor phase I. Lennard-Jones fluid. J. Chem. Phys. 109, 8451–8462 (1998). 10.1063/1.2712436 17411136

[b28] Wolde P. R. & Frenkel D. Computer simulation study of gas-liquid nucleation in a Lennard-Jones system. *J*. Chem. Phys. 109, 9901–9918 (1998).

[b29] Diemand J., Angélil R., Tanaka K. K. & Tanaka H. Large scale molecular dynamics simulations of homogeneous nucleation. J. Chem. Phys. 139, 074309 (2013). 23968094 10.1063/1.4818639

[b30] Yasuoka K., Gao G. T. & Zeng X. C. Molecular dynamics simulation of supersaturated vapor nucleation in slit pore. J. Chem. Phys. 112, 4279–4285 (2000).

[b31] Toxværd S. Molecular dynamics simulation of heterogeneous nucleation at a structureless solid surface. J. Chem. Phys. 117, 10303–10310 (2002).

[b32] Xu W., Lan Z., Peng B. L., Wen R. F. & Ma X. H. Effect of surface free energies on the heterogeneous nucleation of water droplet: a molecular dynamics simulation approach. J. Chem. Phys. 142, 054701 (2015). 25662654 10.1063/1.4906877

[b33] Niu D. & Tang G. H. The effect of surface wettability on water vapor condensation in nanoscale. Sci. Rep. 6, 19192 (2016). 26754316 10.1038/srep19192 PMC4709718

[b34] Niu D. & Tang G. H. Static and dynamic behaviour of water droplet on solid surfaces with pillar-type nanostructures from molecular dynamics simulation. Int. J. Heat Mass Transfer 79, 647–654 (2014).

[b35] Stillinger F. H. Rigorous basis of the frenkel-band theory of association equilibrium. J. Chem. Phys. 38, 1486–1494 (1963).

[b36] Xue L., Keblinski P., Phillpot S. R., Choi S. U. S. & Eastman J. A. Two regimes of thermal resistance at a liuid-solid interface. J. Chem. Phys. 118, 337–339 (2003).

[b37] Sun J., Wang W. & Wang H. S. Dependence of nanoconfined liquid behavior on boundary and bulk factors. Phys. Rev. E 87, 023020 (2013). 10.1103/PhysRevE.87.023020 23496623

[b38] Kalikmanov V. I. Nucleation theory (Springer, 2013).

[b39] Loeffler T. D. & Chen B. Surface induced nucleation of a Lennard-Jones system on an implicit surface at sub-freezing temperatures: a comparison with the classical nucleation theory. J. Chem. Phys. 139, 234707 (2013). 24359386 10.1063/1.4848737

[b40] Miljkovic N. . Jumping-droplet-enhanced condensation on scalable superhydrophobic nanostructured surfaces.Nano Lett. 13, 179–187 (2013). 23190055 10.1021/nl303835d

[b41] Rykaczewski K. . Dropwise condensation of low surface tension fluids on omniphobic surfaces. Sci. Rep 4, 04158 (2014). 10.1038/srep04158 PMC3942741 24595171

[b42] Wu Y. & Zhang C. Analysis of anti-condensation mechanism on superhydrophobic anodic aluminum oxide surface. Appl. Therm. Eng. 58, 664–669 (2013).

[b43] Yi P., Poulikakos D., Walther J. & Yadigaroglu G. Molecular dynamics simulation of vaporization of an ultra-thin liquid argon layer on a surface. Int. J. Heat Mass Transfer 45, 2087–2100 (2002).

[b44] Maruyama S. Molecular dynamics method for microscale heat transfer in Advances in numerical heat transfer, Vol. 2, (eds Minkowycz W. J. & Sparrow E. M.) Ch. 6, 189–226 (CRC Press, 2000).

[b45] Thompson P. A. & Robbins M. O. Shear flow near solids: Epitaxial order and flow boundary conditions. Phys. Rev. A 41, 6830 (1990). 9903096 10.1103/physreva.41.6830

[b46] Thompson P. A. & Troian S. M. A general boundary condition for liquid flow at solid surfaces. Nature 389, 360–362 (1997).

[b47] Delgado-Buscalioni R. & Coveney P. V. USHER: an algorithm for particle insertion in dense fluids. J. Chem. Phys. 119, 978–987 (2003).

[b48] Rapaport D. C. The art of molecular dynamics simulation (Cambridge University Press, 2004).

[b49] Allen M. P. & Tildesley D. J. Computer simulation of liquids (Clarendon Press, 1989).

