# Supplementary material for: On the onset of surface condensation: formation and transition mechanisms of condensation mode
Source: Sci Rep. 2016 Aug 2;6:30764. doi: 10.1038/srep30764 (PMC4969758; doi:10.1038/srep30764)
Supplement: Supplementary Information [file srep30764-s1.docx]

On the onset of surface condensation: Formation and transition mechanisms of condensation modes

# Qiang Sheng, Jie Sun, Qian Wang, Wen Wang and Hua Sheng Wang

**Video Legends**

**Supplementary Video S1:** Snapshots of condensation on the surface with $\beta=0.15$. Simulation system size: $l_{x}\times l_{y}\times l_{z}=47.2\text{ }\sigma\times48.0\text{ }\sigma\times92.0\text{ }\sigma$. Only the lower half of simulation system is shown. The upper left panel shows the 45° tilt angle view. The upper right panel shows the side view. The lower left panel shows the front view. The lower right panel shows the top view.

**Supplementary Video S2:** Snapshots of condensation on the surface with $\beta=0.30$. Simulation system size: $l_{x}\times l_{y}\times l_{z}=47.2\text{ }\sigma\times48.0\text{ }\sigma\times92.0\text{ }\sigma$. Only the lower half of simulation system is shown. The upper left panel shows the 45° tilt angle view. The upper right panel shows the side view. The lower left panel shows the front view. The lower right panel shows the top view.

**Supplementary Video S3:** Snapshots of condensation on the surface with $\beta=0.45$. Simulation system size: $l_{x}\times l_{y}\times l_{z}=47.2\text{ }\sigma\times48.0\text{ }\sigma\times92.0\text{ }\sigma$. Only the lowermost 1/6 of simulation system is shown. The upper left panel shows the 45° tilt angle view. The upper right panel shows the side view. The lower left panel shows the front view. The lower right panel shows the top view.

**Supplementary Video S4:** Snapshots of condensation on the surface with $\beta=0.45$ and larger area. Simulation system size: $l_{x}\times l_{y}\times l_{z}\text{ = 92.7 }\sigma\times\text{93.0 }\sigma\times\text{183.0 }\sigma$. Only the lower half of simulation system is shown. The upper left panel shows the 45° tilt angle view. The upper right panel shows the side view. The lower left panel shows the front view. The lower right panel shows the top view.

**Supplementary Video S5:** Snapshots of condensation on the surface with $\beta=0.75$. Simulation system size: $l_{x}\times l_{y}\times l_{z}=47.2\text{ }\sigma\times48.0\text{ }\sigma\times92.0\text{ }\sigma$. Only the lower half of simulation system is shown. The upper left panel shows the 45° tilt angle view. The upper right panel shows the side view. The lower left panel shows the front view. The lower right panel shows the top view.
