# Supplementary figures and images for: On the onset of surface condensation: formation and transition mechanisms of condensation mode
Source: Sci Rep. 2016 Aug 2;6:30764. doi: 10.1038/srep30764 (PMC4969758; doi:10.1038/srep30764)

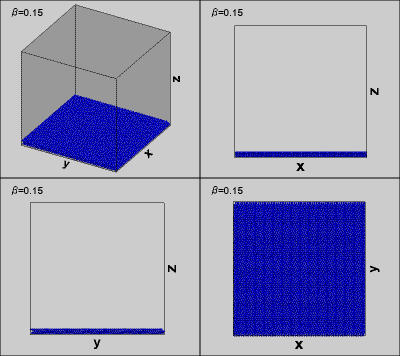

Supplement: Supplementary Information [file srep30764-s2.gif]

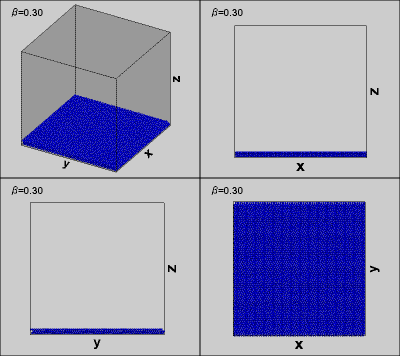

Supplement: Supplementary Information [file srep30764-s3.gif]

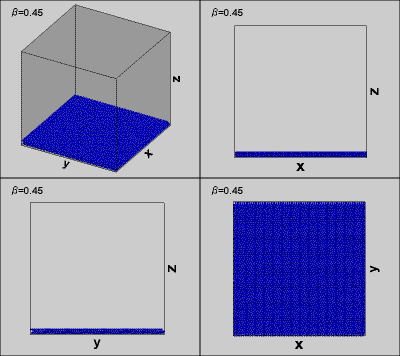

Supplement: Supplementary Information [file srep30764-s4.gif]

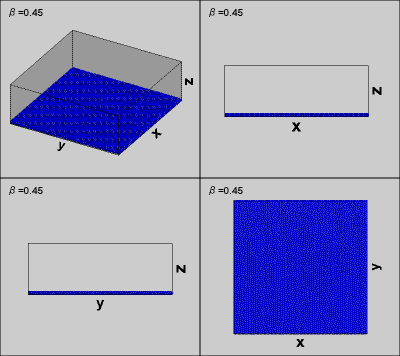

Supplement: Supplementary Information [file srep30764-s5.gif]

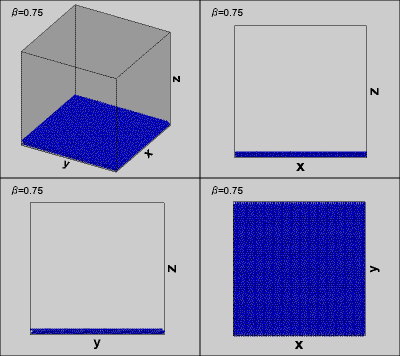

Supplement: Supplementary Information [file srep30764-s6.gif]
